# The making of a miscreant: tobacco smoke and the creation of pathogen-rich biofilms

**DOI:** 10.1038/s41522-017-0033-2

**Published:** 2017-10-24

**Authors:** Samir A. Shah, Sukirth M. Ganesan, Saradhadevi Varadharaj, Shareef M. Dabdoub, John D. Walters, Purnima S. Kumar

**Affiliations:** 1 Division of Periodontology, College of Dentistry, The Ohio State University, 4111 Postle Hall. 305, W 12th Avenue, Columbus, OH 43210 USA; 2 Division of Biosciences, College of Dentistry, The Ohio State University, Columbus, OH USA; 3 Davis Heart Lung Research Institute, The Ohio State University, Columbus, OH USA; 4 Present Address: South Jersey Periodontics, New Jersey, USA; 5 Present Address: Medical Safety and Surveillance, Abbott Laboratories, Columbus, OH USA

## Abstract

We have previously reported that oral biofilms in clinically healthy smokers are pathogen-rich, and that this enrichment occurs within 24 h of biofilm formation. The present investigation aimed to identify a mechanism by which smoking creates this altered community structure. By combining in vitro microbial–mucosal interface models of commensal (consisting of *Streptococcus oralis, Streptococcus sanguis, Streptococcus mitis, Actinomyces naeslundii, Neisseria mucosa* and *Veillonella parvula)* and pathogen-rich (comprising *S.oralis, S.sanguis, S.mitis, A.naeslundii, N.mucosa* and *V.parvula*, *Fusobacterium nucleatum, Porphyromonas gingivalis, Filifactor alocis, Dialister pneumosintes, Selenonomas sputigena, Selenominas noxia, Catonella morbi, Parvimonas micra* and *Tannerella forsythia)* communities with metatranscriptomics, targeted proteomics and fluorescent microscopy, we demonstrate that smoke exposure significantly downregulates essential metabolic functions within commensal biofilms, while significantly increasing expression of virulence genes, notably lipopolysaccharide (LPS), flagella and capsule synthesis. By contrast, in pathogen-rich biofilms several metabolic pathways were over-expressed in response to smoke exposure. Under smoke-rich conditions, epithelial cells mounted an early and amplified pro-inflammatory and oxidative stress response to these virulence-enhanced commensal biofilms, and a muted early response to pathogen-rich biofilms. Commensal biofilms also demonstrated early and widespread cell death. Similar results were observed when smoke-free epithelial cells were challenged with smoke-conditioned biofilms, but not vice versa. In conclusion, our data suggest that smoke-induced transcriptional shifts in commensal biofilms triggers a florid pro-inflammatory response, leading to early commensal death, which may preclude niche saturation by these beneficial organisms. The cytokine-rich, pro-oxidant, anaerobic environment sustains inflammophilic bacteria, and, in the absence of commensal antagonism, may promote the creation of pathogen-rich biofilms in smokers.

## Tobacco: How smoking promotes harmful biofilms

Tobacco smoke inhibits the metabolism of beneficial bacteria in biofilms, while activating specific genes in pathogenic bacteria. This suggests a mechanism to explain how smoking quickly leads to the formation of damaging biofilms in the mouth and respiratory tract. Purnima Kumar and colleagues at Ohio State University, USA studied the effect of tobacco smoke on cultured biofilms used to model those that form on mucous membranes. They detected specific and varied changes in the activity of genes, proteins and metabolism that allowed pathogenic bacteria to displace beneficial “commensal” bacteria. The research suggests the transition toward pathogen-rich biofilms may contribute to the health effects of smoking by causing increased inflammation of mucous membranes and the production of damaging oxidant chemicals. Further research should investigate the chemical constituents of smoke responsible for these effects.

## Introduction

It is well known that an intact epithelial barrier plays a critical role in maintaining the health of mucosal surfaces, both by preventing ingress of bacteria and by secreting immuno-modulatory signaling molecules.^1,2^ Evidence is now emerging that host-associated bacterial biofilms play a similarly important role in maintaining mucosal health.^3,4^ Commensal bacteria prevent pathogen colonization in different habitats by saturating these niches and educating the immune system to differentiate between “friend and foe”. A biofilm that elicits a low immune response is seen by the epithelium as health-compatible and as pathogenic when it has a high antigenic load.^5–7^ Equilibrium between these biofilms and the host immune system is a determinant of health. Disease occurs when the ecosystem is disrupted and triggers a florid host response.^8–10^ For example, in the oral cavity, it has been established that dysbiotic microbial biofilms underlie the etiologies of oral cancer, caries, and periodontal diseases.^11–15^

Smokers are at especially high risk for oral cancer and periodontitis, with a 16-fold increase in odds for developing extensive and severe disease when compared to nonsmokers.^16^ We have previously demonstrated that smokers not only have pathogen-rich, commensal-poor biofilms in disease, but also that this dysbiosis is established long before the onset of clinical disease.^17,18^ Moreover, this pathogen enrichment occurs very early during the colonization of the biofilm.^19^ However, the mechanism underlying the commensal clearance and pathogen acquisition in this high-risk group is not known. Evidence indicates that loss of commensal-rich biofilms decreases the protection offered by them and hence increases susceptibility to disease in the gut, nasopharynx and vagina.^20,21^ Therefore, it is critical to understand the underlying cellular and molecular mechanisms that influence bacterial community assembly in smokers.

Bacterial colonization of biofilms depends on both inter-bacterial and host–bacterial interactions, and evidence is now emerging that the epithelial cells play an active role in assembling mucosa-associated biofilms and in determining their composition.^22^ Several mechanisms have been proposed to explain this role of epithelial cells: expression of surface receptors, secretion of immunomodulatory molecules and provision of nutrients.^10,23^ It is known that smoking alters the phenotype of epithelial cells by affecting immune expression and oxidative stress;^24,25^ it is possible that this may be a possible mechanism by which smoking contributes to altered biofilm colonization. Hence, we sought to explore the effect of smoking on oral host–bacterial interactions, using a mucosal–microbial interface model to mimic the subgingival environment.

## Results

### ‘MiMIC’ry

We initially investigated the ability of microbial–mucosal interface construct (MiMIC) to recapitulate the subgingival epithelial–microbial interface. Visually, the in vitro biofilms demonstrated characteristics similar to those previously described in vivo subgingival biofilms,^26^ with a multi-layered structure, fibril-mediated coaggregation between colonizers, channel-like structures separating the micro-colonies, and a sequential overlaying of the secondary colonizers (bacillary, cocco-bacillary shapes) over the primary colonizers (cocci) (Supplemental Fig. 1a i, ii). Compositionally, the abundances of the specific species in the biofilms were similar to those reported from in vivo biofilms in periodontal health and disease^27,28^ (Supplemental Fig. 1b). The immune mediators secreted by the epithelial monolayer also demonstrated significantly different concentrations when challenged with the commensal or pathogen-rich biofilms (Supplemental Fig. 1c). The concentrations of these mediators were comparable to those previously observed in periodontal health and disease.^29^

### Host–bacterial interactions

**Fig. 1:**
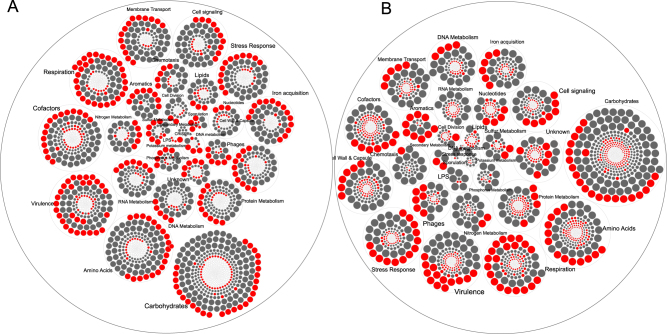
Hierarchical circle packing plot of transcriptional activity in commensal (**a**) and pathogen-rich (**b**) biofilms. Each circle represents a gene and is sized by log_2_ fold change. Genes are grouped based on their functional roles based on SEED classification. Genes that were significantly over-expressed (log_2_ fold change >2, *p* < 0.05, FDR-adjusted Wald test) in a smoke-rich environment when compared to controls are in red, while those that were under-expressed following smoke conditioning are shown in gray. White circles indicate genes whose change in expression did not meet the above criteria. The data used in creating this Figure are shown in Supplementary Table 1

Exposure to cigarettes smoke led to downregulation of 1465 genes belonging to 459 functional families and upregulation of 469 genes contributing to 88 functions in commensals (Fig. 1 and Supplemental Table 1). By contrast, in pathogen-rich biofilms, smoke conditioning led to downregulation of 1058 genes (corresponding to 249 functions) and upregulation of 1497 genes that contributed to 252 functions. In commensal biofilms, significant downregulation of genes relating to basic metabolic pathways (carbohydrate, protein, DNA and RNA metabolisms, respiration, stress response and membrane transport) and upregulation of certain virulence (LPS, flagella and capsule synthesis) and fermentative pathways were observed. In pathogen-rich communities, smoke exposure downregulated genes contributing to aerobic carbohydrate metabolism, LPS, flagella and upregulated fermentative pathways, stress response and iron acquisition.

**Fig. 2:**
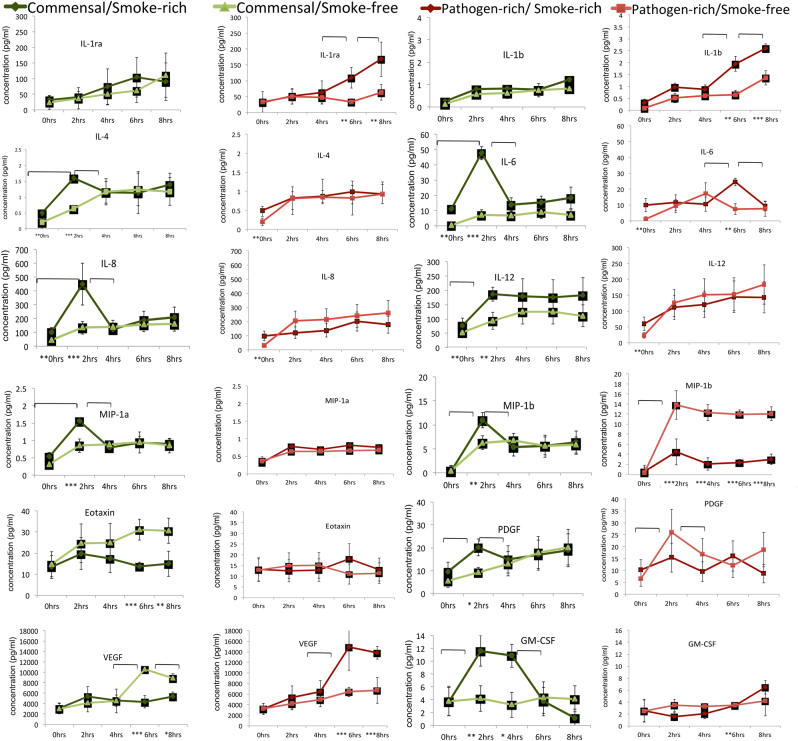
Cytokine released by OKF6-TERT cells in response to biofilm challenge. Levels of selected cytokines in response to commensal (greens) and pathogen-rich (reds) biofilms in smoke-free and smoke-rich environments are shown. Data represent means of six replicates, with standard deviation bars. (* *p* < 0.05, **p < 0.01, ***p < 0.001, between-group differences, brackets represent *p* < 0.01, repeated measures ANOVA)

**Fig. 3:**
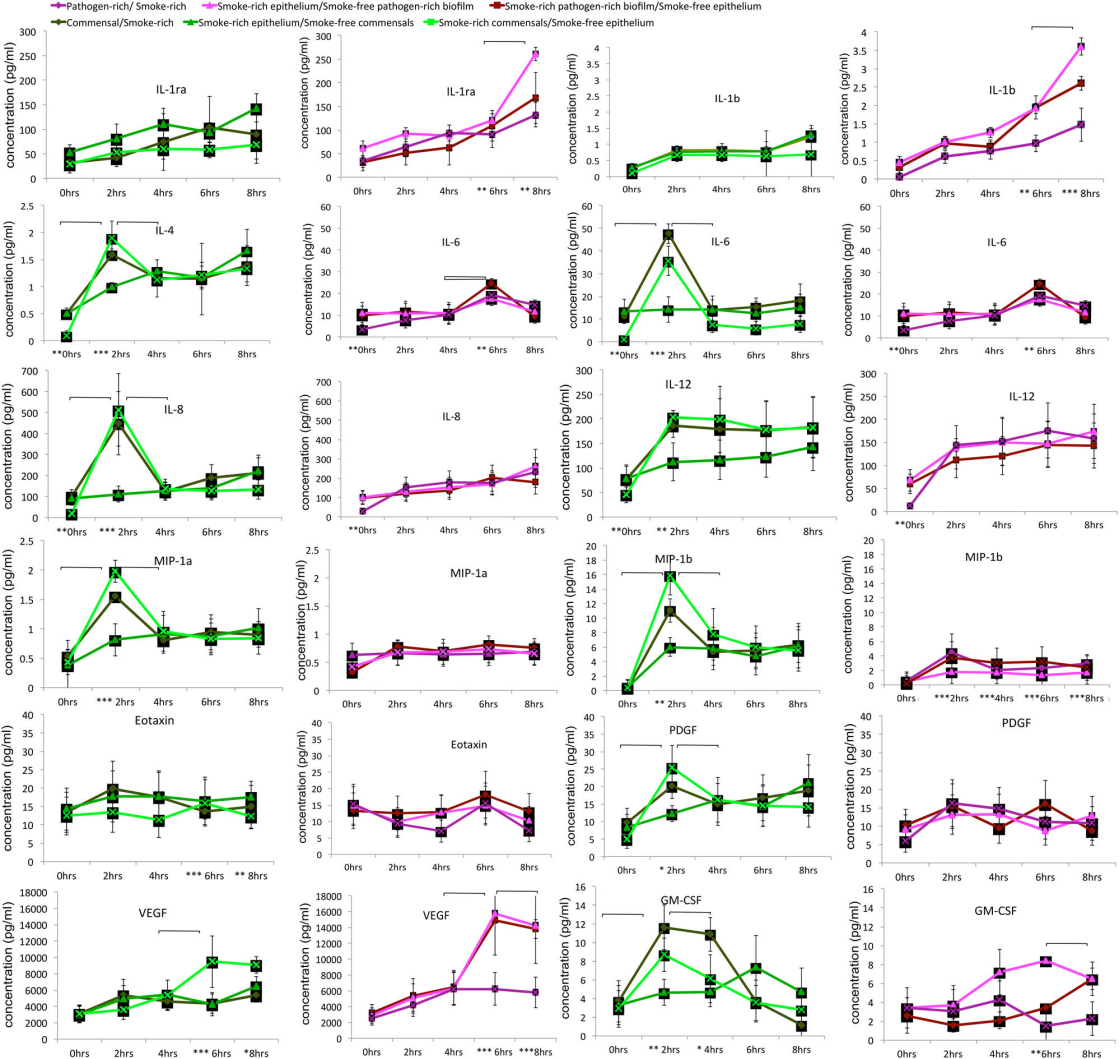
Cytokine released by smoke-conditioned OKF6-TERT cells in response to smoke-conditioned and smoke-free biofilm challenge. Levels of selected cytokines in response to commensal (*greens*) and pathogen-rich (*reds*) biofilms in smoke-free and smoke-rich environments are shown. Data represent means of six replicates, with standard deviation *bars*. (* *p* < 0.05, **p < 0.01, ***p < 0.001, between-group differences, brackets represent *p* < 0.01, repeated measures ANOVA)

We then examined the responses of the epithelial cells to these commensal and pathogen-rich biofilms at 2,4,6 and 8 h in smoke-free and smoke-rich environments (Fig. 2). Early (2 h) increases in IL-4, IL-6, IL-8, IL-12, GM-CSF, MIP-1α, MIP-1β and PDGF and late (6 and 8 h) decreases in Eotaxin and vascular endothelial growth factor (VEGF) were observed in response to commensal biofilms in a smoke-rich environment (*p* < 0.05, repeated measures analysis of variance (ANOVA)). Smoke conditioning also led to late (6 and 8 h) increases in IL-1β, IL-6 and VEGF and sustained decrease in MIP-1b response to pathogen-rich biofilms. Since cytokine production can be impacted both by the type of bacterial challenge as well as smoke exposure, we then investigated if these effects were attributable to a smoke-induced hyper-inflammatory response or to a bacterial challenge or to both. When the MiMIC was recreated using either smoke-conditioned biofilm or epithelium (i.e., smoke-conditioned epithelium challenged with smoke-free biofilms and vice versa), it was seen that the responses of smoke-free epithelium to smoke-rich commensal biofilms were significantly similar to those of the smoke-rich overall environment with respect to several inflammatory mediators (*p* < 0.05, ANOVA, Fig. 3).

**Fig. 4:**
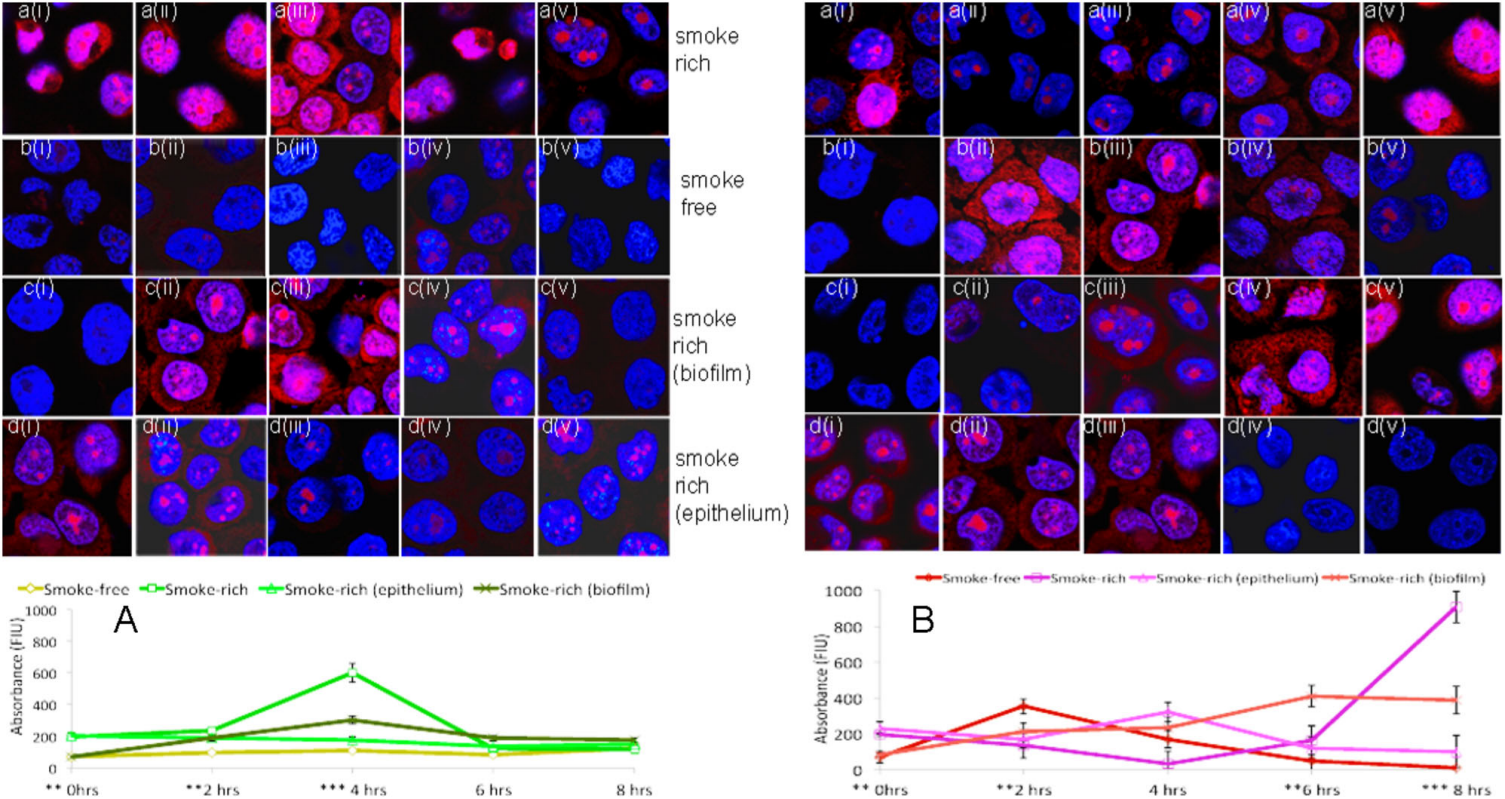
Intracellular ROS production in response to commensal and pathogen-rich biofilms in smoke-free and smoke-rich environments. Figure 4a shows ROS production in response to commensal biofilm challenge, while Fig. 4b shows ROS production in response to pathogen biofilm challenge. Data represent means of six replicates, with standard deviation bars. i–v indicate sample micrographs at 0, 2, 4, 6 and 8 h respectively, while **a–d** indicates the environment at the same time points (*p < 0.05, **p < 0.01, ***p < 0.001, repeated measures ANOVA)

Smoke exposure, by itself, led to a higher reactive oxygen species (ROS) generation by epithelial in the absence of a bacterial challenge (0 h, Fig. 4). There was also a significantly greater ROS response to commensal biofilms at 2 and 4 h in a smoke-rich environment. Moreover, this increase was evident when smoke-free epithelial cells were challenged with smoke-rich commensal biofilms. On the other hand, epithelial cells responded with significantly greater ROS generation at 2 h in response to pathogens in a smoke-free environment. However, in a smoke-rich environment, this response was delayed to 8 h (*p* < 0.05, repeated measures ANOVA).

Since both transcriptional and proteomic activity depend on live cells, we measured viability bacterial biofilms and epithelial cells over the 8-h period at different concentrations of cigarette smoke extract (CSE). Commensal biofilms demonstrated a 20% loss in viability at 2 h, 50% at 8 h (*p* < 0.05, repeated measures ANOVA, Supplemental Fig. 2a). This was observed for all concentrations of CSE (all-or-none pattern). Pathogen-rich biofilms, on the other hand, demonstrated a dose response in viability, with 2 and 5% CSE leading to a significant decrease in viability at 8 h when compared to the lower concentrations (*p* < 0.05, repeated measures ANOVA, Supplemental Fig. 2b). Pathogen biofilms did not demonstrate a significant loss of viability in 1% CSE during the 8-h observation period. There was no significant loss of epithelial viability during the observation period with any concentration of CSE (Supplemental Fig. 2c, d).

Previous investigations have used single bacterial species to examine host–bacterial interactions.^30–32^ To test the hypothesis that single species can be used as surrogates for multi-species biofilms, *Streptococcus mitis* biofilms were compared to the multi-species commensal biofilms, while *P. gingivalis* biofilms were compared to multi-species pathogen-rich biofilms (Supplemental Fig. 3). The responses of single species were significantly different from the multi-species biofilms. Greater cytokine responses (notably IL-1B, IL-4, IL-6, PDGF, MIP-1B, GM-CSF) were observed at later time points (6 and 8 h) to *P.gingivalis* biofilms when compared to multi-species biofilms both in the presence and absence of smoke. Cytokine responses to *S.mitis* biofilms did not demonstrate a consistent pattern when compared to multi-species commensal biofilms either in the presence or absence of smoke.

## Discussion

This study sought to investigate the mechanism by which smoking creates commensal-poor, pathogen-rich communities in different mucosal niches. Although the oral cavity is one of the most accessible ecosystems in the human body, there are several difficulties associated with studying this process in vivo. Repeatedly sampling the same site over short spans of time alters the quality and quantity of the plaque biofilm and gingival crevicular fluid, and the results may not accurately reflect host–bacterial interactions. Hence, an in vitro model of the microbial–mucosal interface was used in this study to overcome these issues. This is a modification of the original Zurich 10-species model,^33–36^ and the present investigation attests to the versatility of the model in allowing the incorporation of customized bacterial consortia into the biofilm. We acknowledge that is a highly simplified model, and does not fully replicate the intricate dynamics between a polymicrobial biofilm and a complex multi-cellular immuno-inflammatory apparatus, however, our data demonstrates that it recapitulates the functional profiles of the subgingival biofilm, as well as the innate immune responses of the sulcular epithelium. Recent studies using proteomics and transcriptomics of fibroblastic responses to these biofilms also serve to validate the ability of this system to mimic events in the subgingival sulcus.^36,37^

The time points selected were based on evidence that innate immune responses occur within 6–8 h of bacterial colonization,^38^ as well as on the sequence of bacterial colonization described by Diaz et al, with initial colonization beginning at 2 h and a climax community at 8 h.^39^ Also, preliminary studies revealed greater than 25% loss of viability of eukaryotic cells following a 12-h pathogen challenge or 24-h smoke exposure. One percent CSE was used to test the effect of smoking because the contents of the CSE produced in this setting reflect the plasma nicotine concentrations of 10-pack year smokers.^40,41^ Previous investigations have reported that the nutritional conditions within the MiMIC affect the transcriptional activity of the cells.^33^ In the present investigation, while it is possible that the nutritional environment created by the media may be responsible for some of the transcriptional and proteomic readouts, however, since the conditions were the same across groups, the effects are comparable.

Epithelial cells are the first responders to bacterial presence in all mucosal niches; they are part of an innate immune apparatus that secretes chemo-attractive molecules to initiate an inflammatory response.^1,42^ The cross talk between oxidative stress and pro-inflammatory immune response plays a critical role in the initiation and progression of several diseases. More importantly, oxidative stress plays a major role in the pathogenesis of smoking-related diseases.^43^ Since virtually nothing is known about oxidative stress responses to commensal and pathogenic bacterial communities, we investigated the ability of these biofilms to induce intra-cellular ROS.

Our results demonstrate that under normal circumstances, the inflammatory and stress responses to a commensal biofilm are more muted than to a pathogen-rich community. The commensal biofilms also elicited high levels of IL-10 from the epithelial cells. Evidence from in vivo studies indicates that commensals suppress TLR4 activation through the IL-10 pathway.^8^ Thus, our results are in line with several studies that demonstrate a differential cytokine expression to pathogenic and non-pathogenic triggers^5,44,45^ and serve to validate the present study.

By contrast, in a smoke-rich environment, epithelial cells mounted a robust and early pro-inflammatory response to commensal biofilms (Figs. 2 and 3). This was also seen in the amount of ROS generated by the epithelial cells (Fig. 4a). The early response to pathogen-rich biofilms, by comparison, was highly subdued. These findings corroborate several lines of evidence in the literature suggesting that the innate immune response to pathogens is abrogated in smokers.^24,46^ Since dead bacteria are incapable of producing an important recognition molecule, vita-PAMP (viability-associated pathogen-associated molecular patterns), they elicit a significantly lower innate immune response.^47^ In order to investigate if this dampened response to pathogens was caused by cellular death, we examined both biofilm and epithelial viability over the duration of the study. Surprisingly, smoke-treated commensals, and not pathogens, demonstrated an early and significant loss of viability. This commensal death was observed following the inflammatory and pro-oxidant surge at 2 h. In the oral ecosystem, niche saturation by pioneer organisms plays an important role in creating colonization resistance to pathogens. It is possible that this early commensal death affects downstream events in biofilm formation, and may play a central role in creating the pathogen enrichment that was previously reported by our group and others.

In the present investigation, smoke-conditioned epithelium secreted greater amounts of pro-inflammatory mediators even prior to bacterial challenge (0 h, Fig. 2), corroborating previous evidence that smoking constitutively increases immune responses in these cells.^48,49^ Since epithelial cells play an important role in acquiring and assembling mucosal microbial communities, we used two approaches to investigate the importance of this constitutive inflammation on commensal depletion. Initially, we challenged smoke-conditioned epithelial cells with smoke-free biofilms and vice versa (Fig. 2). The immune responses of smoke-free epithelial cells to smoke-conditioned commensal biofilms closely mimicked those of the smoke-rich overall environment, suggesting that the effects of smoking on the microbiome contribute largely to its detrimental effects on host–bacterial interactions. To investigate what these effects on the biofilm are, we examined the effects of smoking on gene expression within commensal and pathogen-rich biofilms. The most striking functional shift was a downregulation of primary metabolic functions within commensal biofilms and upregulation in pathogen-rich communities following 2 h of smoke conditioning. Importantly, fermentative pathways for energy acquisition were upregulated in both biofilms. We have previously demonstrated that energy efficiency is a central hallmark of health-compatible biofilms.^50^ Fermentation yields several short chain fatty acids (SCFA) such as butyrate, propionate and isobutyrate, which impair epithelial cell functions^51^ while increasing apoptosis and necrosis.^52^ These SCFAs have also been strongly associated with periodontitis.^53,54^ In vivo, such alterations in primary metabolic pathways could have important implications for metabolic partnerships and the development of the climax microbial community.

Glutathione is an important redox-buffering compound that protects bacterial cells from osmotic stress, electrophiles and oxidative stress by scavenging reactive oxygen.^55,56^ The present investigation demonstrates a marked reduction in glutathione mediated stress response in commensals, but not pathogens following smoke exposure (Supplemental Table 1). In the presence of amplified ROS expression by epithelial cells, this diminished capability can only be detrimental to commensal survival in smokers.

Iron is important for bacterial survival since it facilitates electron transport, nucleotide synthesis, peroxide reduction and other essential cellular functions. Iron-deprivation leads to expression of several outer membrane proteins, siderophores, hemolysins and toxins, while availability of iron promotes pathogen expansion and cellular invasion.^57–59^ Smoke exposure severely impaired iron acquisition and transport in commensals, and upregulated it in pathogen-rich biofilms. This has important implications for disease, since this might be a contributing factor for tissue invasion of bacteria that has been reported in disease.

Previous studies examining the proteins secreted by gingival epithelial cells in response to pathogen-rich biofilms, especially those populated by red-complex bacteria; have demonstrated a pronounced pro-inflammatory response to these biofilms.^60,61^ One molecule that is common to these red-complex species is Lipid-A, the lipid moiety of LPS, a powerful antigen that elicits a florid pro-inflammatory host response.^62^ Lipid-A synthesis was upregulated in commensal and downregulated in pathogen-rich biofilms conditioned with smoke. This would partly serve to explain the early, exaggerated pro-inflammatory response to commensals and a muted response to pathogen-rich communities in the presence of smoke.

Based on our findings, we propose a model to explain the formation of commensal-poor, pathogen-rich communities in smokers. Normally, biofilm formation begins as a community dominated by commensals^63^; and this niche saturation prevents pathogen colonization.^64,65^ Pathogen colonization is also controlled by host immune system recognition of virulence patterns.^66^ In smokers, a transcriptional shift in the commensal biofilms towards higher virulence combined with a constitutively greater cytokine production triggers a florid pro-inflammatory response from the epithelial cells, leading to early commensal death; and hence precluding niche saturation by these beneficial organisms. On the other hand, in the presence of smoke, these very same virulence signatures are dampened in pathogen-rich biofilms in the early stages, thereby creating a “Trojan Horse”, which the host immune system fails to recognize. Oral pathogens normally thrive in anaerobic, reducing environments; the early pro-inflammatory and oxidative stress response in smokers, along with absence of commensal antagonism, leads to large-scale pathogen colonization, which is fueled and sustained by the florid pro-inflammatory response. The resulting cyclical chain of host-microbe interaction events may ultimately lead to disease.

It is not apparent from this study which of the constituents of cigarette smoke is responsible for the observed effects. The relative contributions of the gaseous and liquid phases of the smoke, as well as the individual constituents, merits further study, especially in light of the fact that electronic nicotine delivery systems, or e-cigs are rapidly gaining popularity among younger smokers.

## Materials and methods

### Microbial–mucosal interface construct (MiMIC)

Cryo-preserved second passage TERT-immortalized human oral keratinocytes (OKF6/TERT-2, purchased from Jim Rheinwald’s laboratory^67^) were thawed and cultured in keratinocyte serum-free medium (Invitrogen, Carlsbad, CA), supplemented with 25 µg/mL of bovine pituitary extract (BPE), 0.2 ng/mL of epidermal growth factor (EGF), 0.3 mM calcium chloride, 100 units/mL penicillin G and 100 µg/mL streptomycin].^67^ Cells were seeded into 6-well Costar tissue culture plates and grown to confluence in Dulbecco’s modified Eagle’s medium-F12 (1:1, vol:vol) (Invitrogen, Carlsbad, Ca, USA) supplemented with 0.2 ng/mL EGF, 25 µg/mL BPE, 1.5 mM L-glutamine, [100 units/mL penicillin G and 100 µg/mL streptomycin] in 5% CO_2_ in air at 37 °C. Prior to biofilm challenge, the test group of cells was conditioned with CSE for 24 h as described below.

Biofilms were developed using the protocol established by Guggenheim et al.^35^ and modified later for subgingival biofilms.^68,69^ Briefly, sterilized, sintered hydroxyapatite discs (Clarkson Chromatography Products, South Williamsport, PA) were incubated in artificial saliva for 24 h to establish a pellicle coat, following which multi-species commensal biofilms were generated by seeding six pioneer species (*Streptococcus oralis, Streptococcus sanguis, Streptococcus mitis, Actinomyces naeslundii, Neisseria mucosa* and *Veillonella parvula)* and incubating under aerobic conditions in a 1:1(vol:vol) mixture of brain–heart Infusion broth (BHI) and artificial saliva. These organisms were selected based on data from our own and Kolenbrander’s work demonstrating their predominance in health-compatible microbiomes.^63^ Pathogen-rich biofilms were created by further seeding the commensal biofilms with an intermediate colonizer (*Fusobacterium nucleatum*), followed 24 h later by *Porphyromonas gingivalis, Filifactor alocis, Dialister pneumosintes, Selenonomas sputigena, Selenominas noxia, Catonella morbi, Parvimonas micra* and *Tannerella forsythia* and incubating under anaerobic conditions for a further 24 h.

The biofilms were overlaid on the OKF6 cells with a 1-mm separation to mimic the microbial–mucosal interface. This was achieved by using 1-mm thick O-rings to separate the discs from the epithelium. Immediately prior to the overlay, spent tissue culture medium was replaced with fresh medium (either with or without CSE, as appropriate). The overlay was incubated in 5% CO_2_ in air at 37 °C for the appropriate time periods.

### Cigarette smoke extraction and conditioning the MiMIC

CSE was prepared immediately before use by bubbling smoke from two 3R4F research cigarettes (9.4 mg tar/0.726 mg nicotine, University of Kentucky) into 20 ml of serum-free KER-SFM and 20 ml of BHI. To maintain consistency of CSE between experiments, optical density measurements were taken at 320 nm, with an optical density of 0.65 representing 100% CSE.^70^ The CSE was diluted to 1% with either BHI or KER-SFM, and the MiMIC conditioned with 1% CSE for 24 h. A volume of 100 μL of the culture medium was aspirated at 2, 4, 6 and 8 h and frozen at −20 °C until further analysis.

### Metatranscriptomics

Total RNA was isolated from the biofilms using the mirVana miRNA isolation kit (Applied Biosystems). Microbial cells were lysed and RNA was extracted by Acid-Phenol: Chloroform and ethanol precipitation and eluted in nuclease-free water. Ribosomal RNA was depleted and mRNA enriched by modified capture hybridization approach. Enriched mRNA served as a template for the polyadenylation reaction and cDNA synthesis. Microbial libraries were clustered on the Illumina HiSeq platform, and 150 bp paired-end sequencing was performed. The Illumina base-calling pipeline was used to process the raw fluorescence images and call sequences. Raw reads with >10% unknown nucleotides or with >50% low quality nucleotides (quality value < 20) were discarded. Microbial transcripts were quality filtered using SolexaQA++, and aligned against the Human Oral Microbiome Database^71^ using DIAMOND.^72^ Aligned sequences were annotated to the KEGG database using Megan 6.^73^ The metagenomic sequence classifier Kraken^74^ was used along with our custom tool, kraken-biom, for taxonomic identification. Analysis and visualization of the distribution of operational taxonomic units was performed using QIIME^75^ and PhyloToAST.^76^ Bioconductor package for R, *DESeq2*, was used to perform differential expression analysis of the annotated microbial transcripts.

### Data availability

All sequences are located in the servers of Argonne National Lab (MG-RAST) and relevant data are available from the authors.

### Cytokine assay

Cytokine analysis was done using a commercially available multiplexed bead-based immunoassay designed to quantitate multiple cytokines.^77^ A panel of 27 cytokines was selected, including Th1 and Th2 cytokines (Interleukin-2 (IL2), Interleukin-12 (IL12), Interferon-γ (INF-γ), Interleukin-1ra (IL-1ra), pro-inflammatory cytokines (Interleukin-1β (IL-1β), Interleukin-6 (IL-6), and granulocyte macrophage colony stimulating factor (GM-CSF)), chemokines (Interleukin-8 (IL-8), interferon gamma-induced protein 10, monocyte chemotactic protein-1, macrophage inflammatory proteins (MIP-1α and MIP-1β), regulated on activation, normal T expressed and secreted and Eotaxin), regulators of T-cells and natural killer cells (interleukin-7 (IL-7) and interleukin-15 (IL-15)), and growth factors (VEGF, plasma-derived growth factor (PDGF)). Briefly, 27 distinct sets of fluorescently dyed beads (Bio-rad laboratories, Inc, Hercules, CA) were conjugated with monoclonal antibodies specific for each cytokine and incubated with 50 μL of supernatant. Biotinylated detection antibody (25 μL) and Streptavidin-phycoerythrin reporter (50 μL) were added sequentially. The level of each cytokine was analyzed by measuring the fluorescence of each bead type as well as the fluorescent signal from the reporter on a Bio-Plex 200 flow cytometric detection system.

### Bacterial viability

Bacterial viability was measured using a BacLight kit (Life Technologies, NY, USA) according to the manufacturer’s instructions. Briefly, the biofilms were incubated in 1.5 mL of 0.3% SYTO 9^**®**^ and propidium iodide and the fluorescence measured at 486 and 520 nm using a Spectral FlowView confocal microscope at ×10 magnification. The ratio of green to red fluorescence was computed and used to determine bacterial viability.

### Epithelial viability

The viability of OKF6-TERT cells was measured using a commercially available lactate dehydrogenase assay (CytoTox-ONE, Promega Corporation, Madison, WI). A volume of 100 μL of the supplied reagent was added to the culture plates with the cells and incubated at 22 °C for 10 min. The reaction was terminated by adding 50 μL of the Stop Solution, and the fluorescence was measured using a fluorometer with an excitation wavelength of 560 nm and an emission wavelength of 590 nm. Positive controls were cells that had been lyzed by freeze-thaw cycles. Cell viability was measured as the ratio of fluorescence between freeze-thaw lyzed (dead) cells and the test groups.

### Reactive oxygen species (ROS)

The level of intracellular superoxide production in OKF6/TERT2 keratinocytes was measured using dihydroethidium (DHE)-derived fluorescence as previously described.^78^ Cells were grown on coverslips in 24-well plates, conditioned with CSE and challenged with biofilms as described earlier. Thirty minutes prior to the allocated time point, 10 μM DHE and 1 μM of Hoescht 33342 (nuclear stain) were added and incubated. At the indicated time points, cells were washed three times with 1× PBS and immediately viewed with an Olympus Spectral FlowView microscope using a ×60 objective, at 405 and 543 nm excitations. Fluorescence intensity, which positively correlates with the amount of O_2_^−^ generation, was measured. High-resolution images were obtained using constant exposure time. A single scan of a new field was used to limit any change in intensity caused by overexposure. The remaining signal in the presence of SOD mimetic Mn(III)tetrakis(4-benzoic acid)porphyrin chloride (MnTBAP) (Alexis Biochemicals, San Diego, CA) was considered to be non-specific (background signal), and therefore was subtracted from all other mean intensities in controls. The fluorescence intensity was quantified using Olympus software. The mean intensity of the fluorescence in a low power field was used for comparison between the groups.

### Statistical analysis

All reactions were carried out in triplicate and assays duplicated. Bioconductor package for R, *DESeq2*, was used to perform differential expression analysis of the annotated microbial transcripts. Phylogenetic trees were visualized using PhyloToAST^76^ and iTol.^79^ Within and between-group comparisons of cytokines and ROS were made using Repeated Measures ANOVA (Tukey HSD) in a Generalized Estimating Equations framework. All analyses were carried out using JMP (Cary, NC) and graphed using the capabilities of R and VORTEX (http://webapp-kumarlab.rhcloud.com/ ^80^).

## Electronic supplementary material

Supplemental figures

Supplemental table 1
