# Supplementary material for: The making of a miscreant: tobacco smoke and the creation of pathogen-rich biofilms
Source: NPJ Biofilms Microbiomes. 2017 Oct 24;3:26. doi: 10.1038/s41522-017-0033-2 (PMC5655325; doi:10.1038/s41522-017-0033-2)
Supplement: Supplementary file 1 — Supplemental figures [file 41522_2017_33_MOESM1_ESM.pdf]

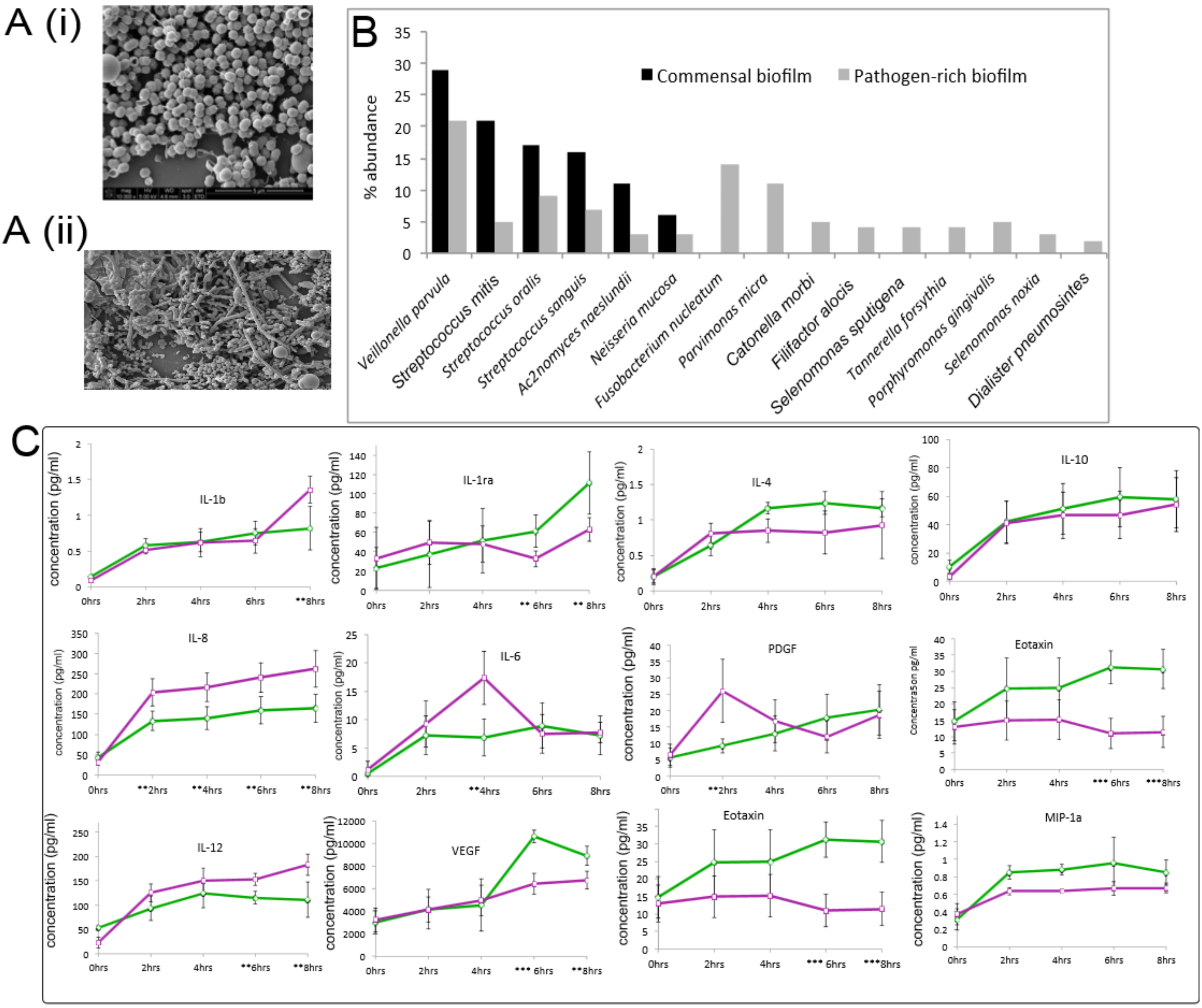

Supplementary Figure 1: Visual, compositional and functional characteristics of *in* vitro biofilms. Scanning electron micrographs of commensal and pathogen-rich biofilms (Figures A (i) and A (ii) respectively) their compositions as determined by 16S sequencing (Figure 1B) and immune responses of the epithelial cells to these biofilms (Figure 1C) are shown.

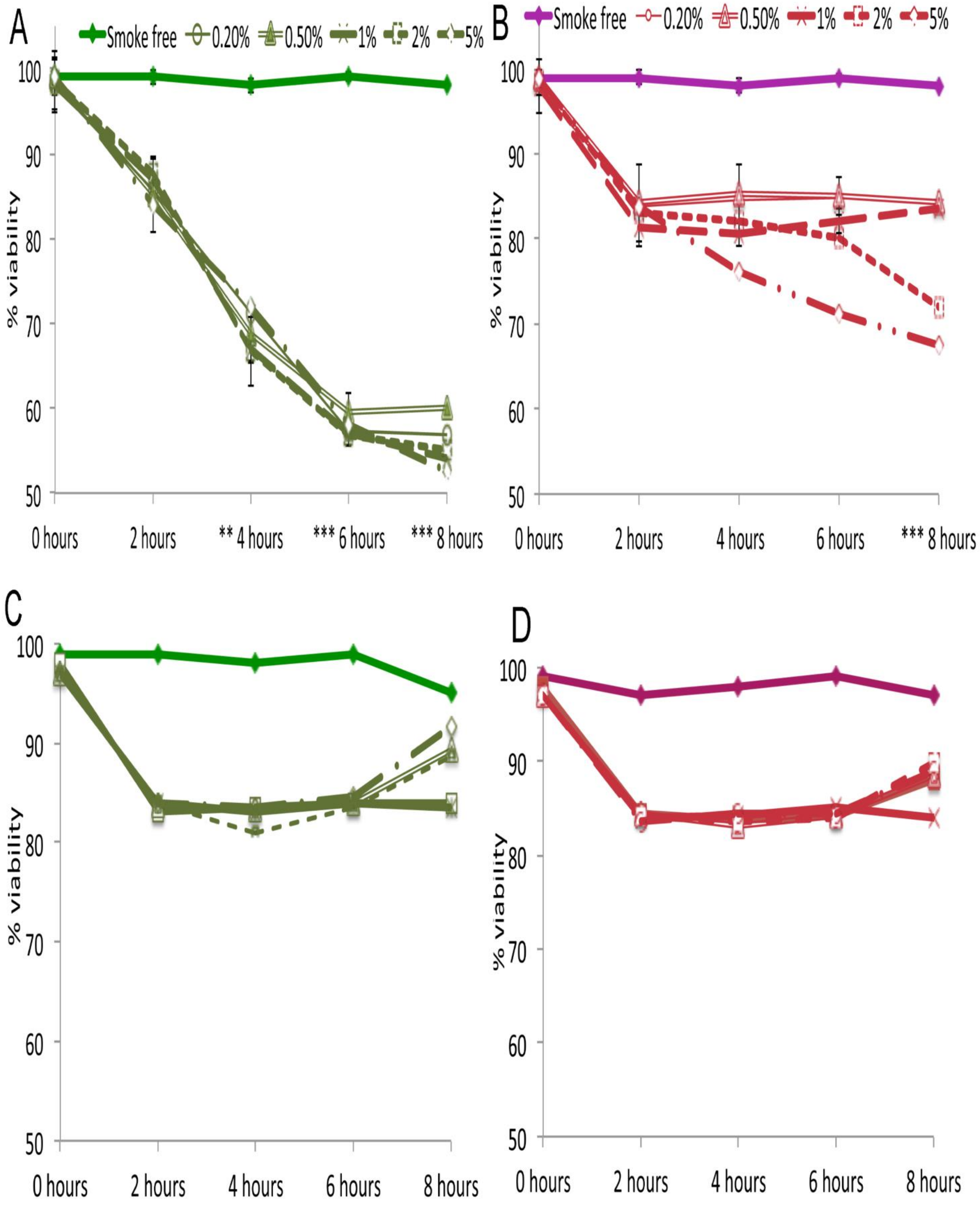

Supplementary Figure 2: Biofilm and epithelial viability over 8 hours in smoke-free and smoke-rich environments. Viability of commensal biofilms in different concentrations of cigarette smoke is shown in Figure 5A, while that of pathogen-rich biofilms shown in Figure 5B. Viability of epithelial cells challenged with commensal biofilms is shown in 5C and epithelial viability in response to smoke concentrations and pathogen challenge is shown in 5D ($^{*}p<0.05$, $^{**}p<0.01$, $^{***}p<0.001$, repeated measures ANOVA).

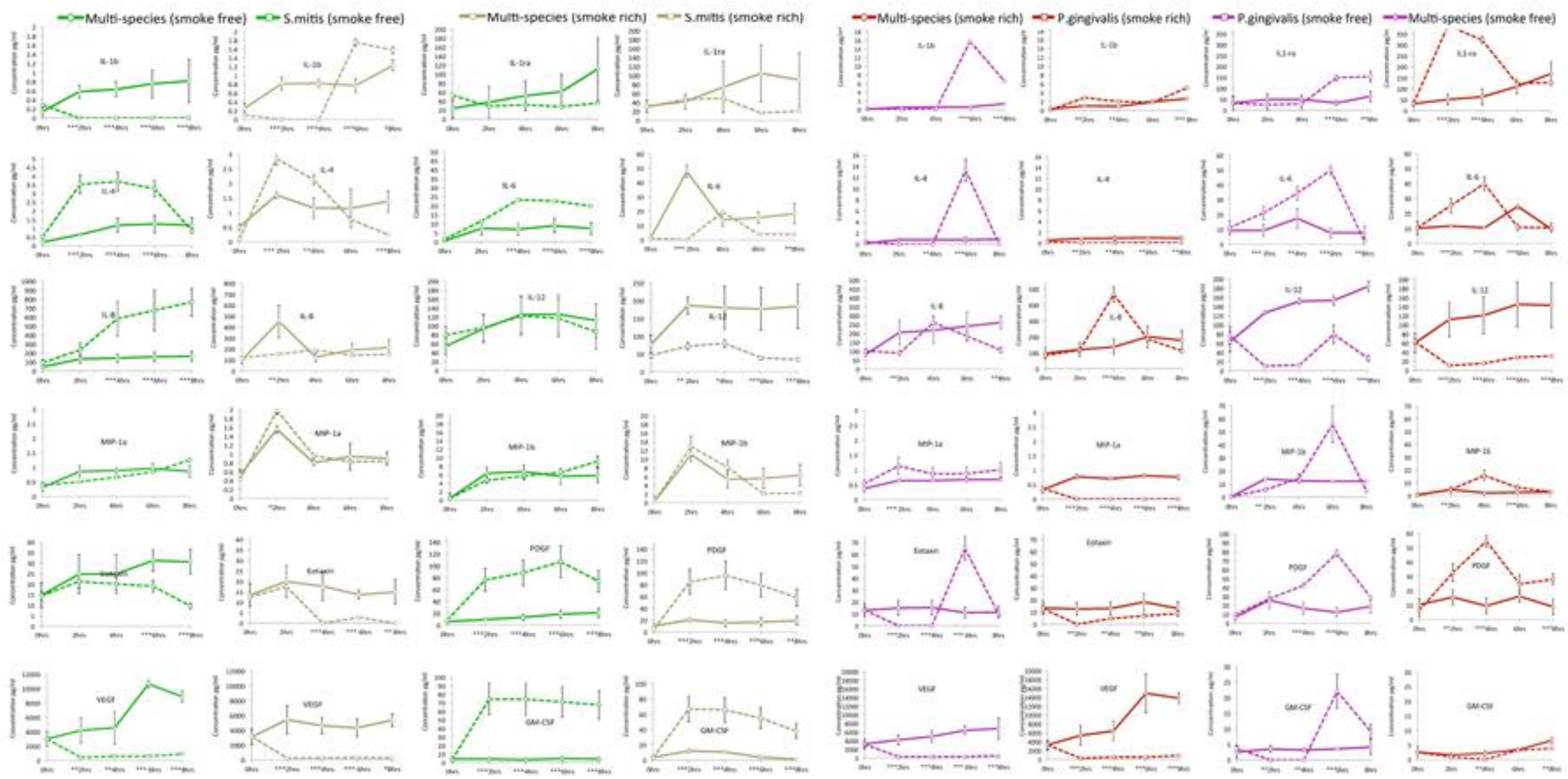

Supplementary Figure 3: Responses of epithelium to single and multi-species biofilms. Responses to *Streptococcus mitis* were compared with commensal biofilms, and responses of *Porphyromonas gingivalis* were compared with pathogen-rich biofilms (* p<0.05, **p<0.01, ***p<0.001, repeated measures ANOVA).
